# Functional analysis of CD44 variants and xCT in canine tumours

**DOI:** 10.1002/vms3.397

**Published:** 2020-11-18

**Authors:** Atsushi Tanabe, Kento Kimura, Hana Tazawa, Takuya Maruo, Masayuki Taguchi, Hiroeki Sahara

**Affiliations:** ^1^ Laboratory of Biology School of Veterinary Medicine Azabu University Kanagawa Japan; ^2^ Veterinary Teaching Hospital Azabu University Sagamihara Japan

**Keywords:** canine, CD44, oxidative stress, radiation, xCT

## Abstract

The cell surface glycoprotein CD44 has various types of splicing variants, which contribute to its multiple distinct cellular functions. Recently, it was reported that the CD44v8‐10 isoform interacts with the system Xc(‐) transporter‐related protein (xCT), and inhibits the accumulation of reactive oxygen species by promoting the synthesis of the antioxidant glutathione in human tumour cells. In this study, we investigated the expression and function of CD44 variants and xCT in canine tumours. From semi‐quantitative reverse transcription polymerase chain reaction analysis, the mRNA expression of the *CD44v8‐10* isoform was observed in canine tumour tissues as well as human cases. The overexpression of CD44v8‐10 may promote the synthesis of glutathione and enhance the resistance to radiation of canine breast tumour cells. Furthermore, canine *xCT* mRNA expression was significantly upregulated in the canine breast tumour tissues as compared to the normal tissues surrounding the tumours. To investigate the function of canine xCT, we treated canine tumour cells with the xCT inhibitor sulfasalazine. Consequently, the sulfasalazine‐treated cells were more sensitive to oxidative stress than the non‐treated cells. Taken together, these results suggested that CD44v8‐10 and xCT play important roles in the therapy resistance of canine tumours as well as human tumours.

## Graphical abstract

CD44v8‐10 contributes H2O2‐ and radio‐resistance of canine breast tumour cells by promoting the production of GSH. A. The expression of CD44s or CD44v8‐10 molecules of these transfectants. B. The H2O2‐resistance of these transfectants. C. The cellular GSH content of CD44 transfectants. D. The radio‐resistance of CD44 transfectants.

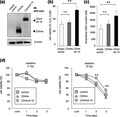

## 1 INTRODUCTION

CD44 is a cell surface molecule that mediates cell adhesion and communication with the extracellular matrix. CD44 has various biological functions, including leukocyte homing, wound healing, cell proliferation and migration, as well as tumour cell growth, invasion and metastasis (Goodison et al., 1999; Ponta et al., 2003). Moreover, CD44 is considered to be a major marker for stem‐like cells in several types of tumours (Zöller, 2011).

The *CD44* gene consists of 20 exons, and exons 6–15 are designated as variant exons v1 to v10. A wide variety of variant isoforms have been generated by alternative mRNA splicing, and these isoforms have multiple distinct cellular functions, such as cell signalling, growth, adhesion and migration (Goodison et al., 1999; Nagano et al., 2013; Ponta et al., 2003; Zöller, 2011). CD44 variant isoform exon 9 (CD44v9) was reported to be upregulated in several tumours, and it is thought to be involved in chemo‐resistance and the recurrence of tumours (Hirata et al., 2013; Horibe et al., 2018; Ishimoto et al., 2011; Ogihara et al., 2019; Wada et al., 2018; Wu et al., 2015; Yoshikawa et al., 2013). CD44v9 is associated with the system Xc(−) transporter‐related protein (xCT), which is a component of the plasma membrane antiporter system xc(−) (Hirata et al., 2013; Horibe et al., 2018; Ishimoto et al., 2011; Ogihara et al., 2019; Wada et al., 2018; Yoshikawa et al., 2013). This system comprises xCT and CD98hc subunits, and it regulates the uptake of cysteine (Huang et al., 2005; Lo et al., 2008). Cysteine is an essential amino acid for the synthesis of glutathione (GSH), which is required for the reduction of intracellular oxidants. Thus, the association between xCT and CD44v9 in the plasma membrane is considered to be an important factor for the intracellular redox system in tumour cells. On the other hand, CD44 standard isoform (CD44s), which contains no variant exons, was revealed to be upregulated in several tumours, such as breast and liver tumours (Asai et al., 2019; Brown et al., 2011; Mima et al., 2012). CD44s‐positive tumour cells show high metastatic ability, whereas their tumourigenicity appears to be dependent on the tumour type (Miwa et al., 2017; Preca et al., 2015).

CD44 is recognized as a cancer stem cell marker in canine breast tumours, (Barbieri et al., 2015; Du et al., 2017; Zhou et al., 2018) and its expression is also increased in canine leukaemia, melanoma and osteosarcoma (Gelain et al., 2014; Guth et al., 2014; Milovancev et al., 2013). Although it is known that the canine *CD44* gene encodes 20 exons, including 10 variant exons, as observed with the human *CD44* gene, the expression pattern and role of CD44 isoforms in canine tumours remain unclear (Milde et al., 1994; Motegi et al., 2018). Here, we investigated the expression and function of CD44s and CD44v in canine tumours.

## 2 MATERIALS AND METHODS

### 2.1 Cell lines and culture conditions

Canine melanoma cell line MCM‐N1 was purchased from DS Pharma Biomedical Co., Ltd. (Osaka, Japan). Canine lung adenocarcinoma cell line CLAC and canine osteosarcoma cell line OS730 were previously established and characterized by a primary canine lung adenocarcinoma (Nemoto et al., 2011) and a primary canine osteosarcoma, (Tanabe et al., 2016) respectively. Canine breast tumour cell line CIMC‐A was provided by Dr. Kikumi Ogihara (Azabu University). CIMC‐A was previously established and characterized by a primary canine inflammatory breast tumour (unpublished data). Canine melanoma cell line CMeC and canine osteosarcoma cell line PoS were provided by Dr. Takayuki Nakagawa (University of Tokyo). CMeC was previously established and characterized by a primary canine skin melanoma, (Inoue et al., 2004) whereas PoS was established from a primary canine osteosarcoma (Kadosawa et al., 1994). Canine osteosarcoma cell line HMPoS and canine mastocytoma cell line CoMS were provided by Dr. Masahiro Okumura (Hokkaido University). HMPoS was previously established and characterized by lung metastatic PoS cells, (Barroga et al., 1999) whereas CoMS were established and characterized by a mast cell tumour of the oral mucosa (Ishiguro et al., 2001).

MCM‐N1, OS730, CIMC‐A and CLAC were cultured in DMEM medium (Sigma‐Aldrich, St Louis, MO, USA), and CMeC, PoS, HMPoS and CoMS were cultured in RPMI1640 medium (Sigma‐Aldrich). Both media were supplemented with 10% foetal bovine serum, 200 unit/ml penicillin (Thermo Fisher Scientific, Waltham, MA, USA), 200 μg/ml streptomycin (Thermo Fisher Scientific) and 2 mM L‐glutamine (Thermo Fisher Scientific), and the cultures were maintained at 37°C in a humidified 5% CO_2_ atmosphere. The canine cancer cell lines used in this study were treated with the mycoplasma remover MC‐210 (Wakenbtech, Kyoto, Japan) before experiments. The canine cancer cell lines were passaged less than 10 times after thawing.

### 2.2 Canine tumour and normal tissue samples

Canine tumour tissues and the normal tissues surrounding the tumours were collected from 39 canine tumour patients who underwent surgery at Taguchi Animal Hospital (Saitama, Japan) between 2010 and 2011. This study was approved by the appropriate ethics committee of Azabu University Veterinary Teaching Hospital, and written informed consent was obtained from all dog owners. The sampling of all tumour tissues and normal tissues surrounding each tumour was performed according to standard surgical resection procedures. The final diagnosis of tumours and normal tissues was determined by histopathological examination, and the tumours were classified based on the pathological diagnosis. Resected tissues were immediately immersed in RNAlater (QIAGEN Inc., Hilden, Germany) and stored at 4°C.

### 2.3 RNA extraction and semi‐quantitative reverse transcription polymerase chain reaction (RT‐PCR)

Total RNA was prepared using an RNeasy Mini Kit (QIAGEN Inc.) according to the manufacturer's instructions, and then reverse‐transcribed to cDNA with a Transcriptor First Strand cDNA Synthesis Kit (Roche Applied Science, Mannheim, Germany). For the analysis of *CD44* splice variants in the canine breast tumour tissues, the normal tissues surrounding the tumours and the canine tumour cell lines, semi‐quantitative RT‐PCR analysis was performed with the following primer sets: *CD44*‐F1 5′‐ CAG TGA AAG GAG CAC TTC GG ‐ 3′ and *CD44*‐R1 5′‐ GAT CCA TGA GTG GTA TGG GAC ‐3′; and *GAPDH*‐F 5′‐ AAC GGG AAG CTC ACT GGC AT ‐3′ and *GAPDH*‐R 5′‐ CTT GAC AAA GTG GTC ATT GAG GG ‐3′.

### 2.4 Quantitative real‐time PCR analysis

Quantitative real‐time PCR analysis of the *GAPDH* and *xCT* (*SLC7A11*) genes was performed using the LightCycler FastStart DNA MasterPLUS SYBR Green I system (Roche Applied Science). The quantitative real‐time PCR was performed with the following primer sets: *xCT*‐F 5′‐ CCA GGT TAT TCT ATG TTG CG ‐3′ and *xCT*‐R 5′‐ CAC CTG GAA AAC TGA GGA A ‐3′; and *GAPDH*‐F 5′‐ AAC GGG AAG CTC ACT GGC AT ‐3′ and *GAPDH*‐R 5′‐ CTT GAC AAA GTG GTC ATT GAG GG ‐3′. PCR amplification of the housekeeping gene *GAPDH* was performed for each sample as a control for sample loading, and to allow normalization among samples. To determine the absolute copy number of the target transcripts, the fragments of the target gene amplified by PCR using the primer sets were ligated into the pGEM‐T‐easy cloning vector (Promega, Madison, WI, USA). The concentrations of these purified plasmids were determined by measuring the absorbance at 260 nm with a spectrophotometer, and the copy numbers were calculated from the concentration of the samples. A standard curve was created by plotting the threshold cycle versus the known copy number for each plasmid template in the dilutions. The copy numbers for all unknown samples were determined according to the standard curve using LightCycler version 3.5.3 (Roche Applied Science). To correct for differences in both RNA quality and quantity between samples, each target gene was first normalized by dividing the copy number of the target by the copy number of *GAPDH*.

### 2.5 Plasmid vector construction and transfection

Using the first‐strand cDNA derived from CIMC‐A as a PCR template, *CD44s* plasmid vector and *CD44v8‐10* plasmid vector were generated by PCR using the following *CD44* primer set: *CD44*‐F2 5´‐ ATG GAC AAG TTG GGG TGG CA‐3′ and *CD44*‐R2 5′‐ CAC CCC AAT CTT CAT ATC CA‐3′. These PCR‐generated DNA fragments containing the coding region of *CD44s* and *CD44v8‐10* were cloned into pCR^TM^ 2.1‐TOPO vector (Thermo Fisher Scientific). The pCR^TM^ 2.1‐TOPO vectors containing *CD44s* or *CD44v8‐10* were digested by Hind III/Not I (*CD44s*) or Kpn I/Not I (*CD44v8‐10*). Subsequently, the samples were ligated into the pcDNA3.1 expression vector (Thermo Fisher Scientific). For transient transfection, 3 × 10^6^ CIMC‐A cells were seeded in a 100‐mm dish, and transfected with 3 μg of *CD44s*, *CD44v8‐10* or control empty vector with the use of the Polyethyleneimine “Max” (Polyscience Inc., Warrington, PA, USA).

### 2.6 H_2_O_2_ and sulfasalazine (SSZ) treatment and MTT assay

Cell survival after H_2_O_2_ treatment was measured by the 3‐(4,5‐dimethylthiazol‐2‐yl)‐2,5‐diphenyltetrazolium bromide (MTT) assay. CIMC‐A cells (3 × 10^6^) were cultured in a 100‐mm dish, and transfected with 3 μg of *CD44s*, *CD44v8‐10* or control empty vector. At 48 hr after the transfection, the cells were trypsinized and counted. Then, 1 × 10^4^ cells were plated in a 96‐well plate, and 1 to 3 mM H_2_O_2_ was added 16 hr later. After 24 hr, 4 mg/ml MTT (Sigma‐Aldrich) in PBS was added (10 μl/well), and the plates were incubated at 37°C for 4 hr. After the incubation, the formazan crystals were dissolved in 100 μl of 0.04 M hydrochloride‐2‐propanol solution, and the absorbance was measured at 570 nm. The cell survival with SSZ treatment was also measured by the MTT assay. CIMC‐A cells (1 × 10^4^) were plated in a 96‐well plate, and 100 μM SSZ was added 16 hr later. After 24 hr, the cells were treated with or without 100 μM H_2_O_2_ for 24 hr, and then the cell viability was measured by the MTT assay.

### 2.7 Exposure to radiation

CIMC‐A cells (3 × 10^6^) were cultured in a 100‐mm dish, and transfected with 3 μg of *CD44s*, *CD44v8‐10* or control empty vector. At 48 hr after the transfection, ionizing radiation at a dose of 0, 1 or 5 Gy was irradiated to the transfected CIMC‐A cells. Radiation was delivered with a linear accelerator radiation machine (Hitachi Medical, Tokyo, Japan). After radiation exposure, the cells were cultured for 1, 3 or 5 days, harvested by trypsinization. Cell viability was measured by the MTT assay and viability rate was calculated as the optical density (OD) value of radiation‐exposed cells/OD value of non‐exposed control cells ×100%.

### 2.8 Measurement of GSH

The intracellular level of GSH was measured with the GSH‐Glo^TM^ Glutathione Assay kit (Promega) according to the manufacturer's instructions. Cells (5 × 10^3^) were suspended in passive lysis buffer, and incubated for 30 min at room temperature. Then, 100 μl of luciferin detection regent was added. After 15 min of incubation at room temperature, the luminescence signal generated in a coupled reaction with firefly luciferase was measured by a Glomax 20/20 Luminometer (Promega).

### 2.9 Western blotting

Anti‐myc antibody was purchased from Santa Cruz Biotechnology, Inc. (Dallas, TX, USA). Anti‐β‐actin antibody was purchased from Sigma‐Aldrich. CIMC‐A cells (3 × 10^6^) were cultured in a 100‐mm dish, and transfected with *CD44s*, *CD44v8‐10* or control empty expression vector. At 48 hr after the transfection, 1 × 10^6^ cells were lysed in 100 μl of lysis buffer (25 mM Tris‐HCl pH 7.4, 150 mM NaCl, 1% Triton X‐100, 1 mM EDTA, 5% glycerol and protease inhibitor cocktail [Roche Applied Science]) for 30 min on ice. Then, 50 μl of 3× sodium dodecyl sulphate sample buffer was added, and the samples were boiled at 100°C for 5 min. Next, the samples were subjected to 8% to 10% sodium dodecyl sulphate‐polyacrylamide gel electrophoresis, then transferred onto a Hybond‐ECL nitrocellulose membrane (Amersham Bioscience, Piscataway, NJ, USA). The transferred antigens on the membrane were detected by Western blotting with anti‐myc or β‐actin antibody.

### 2.10 Statistical analysis

The results represent the mean ± standard deviation (*SD*) values. The Statcel 3 add‐in (OMS Publishing, Saitama, Japan) for Microsoft Excel was used for the statistical analyses. Yates chi‐squared (*χ* ^2^) test was used to analyse the differences in the expression of *CD44* variants between the canine tumours and the normal tissues surrounding the tumours. Statistical analysis of the cytotoxic effects of H_2_O_2_ or radiation treatment was performed using the Student's *t*‐test or two‐way analysis of variance followed by the Tukey–Kramer post hoc test. The *xCT* mRNA expression was compared between the breast tumour tissues and the normal tissues surrounding the tumours using the Wilcoxon rank‐sum test. *p* values <0.05 or <0.01 were considered to be statistically significant.

## 3 RESULTS

### 3.1 Semi‐quantitative RT‐PCR analysis of *CD44* mRNA in canine tumours and normal tissues surrounding the tumours

**FIGURE 1:**
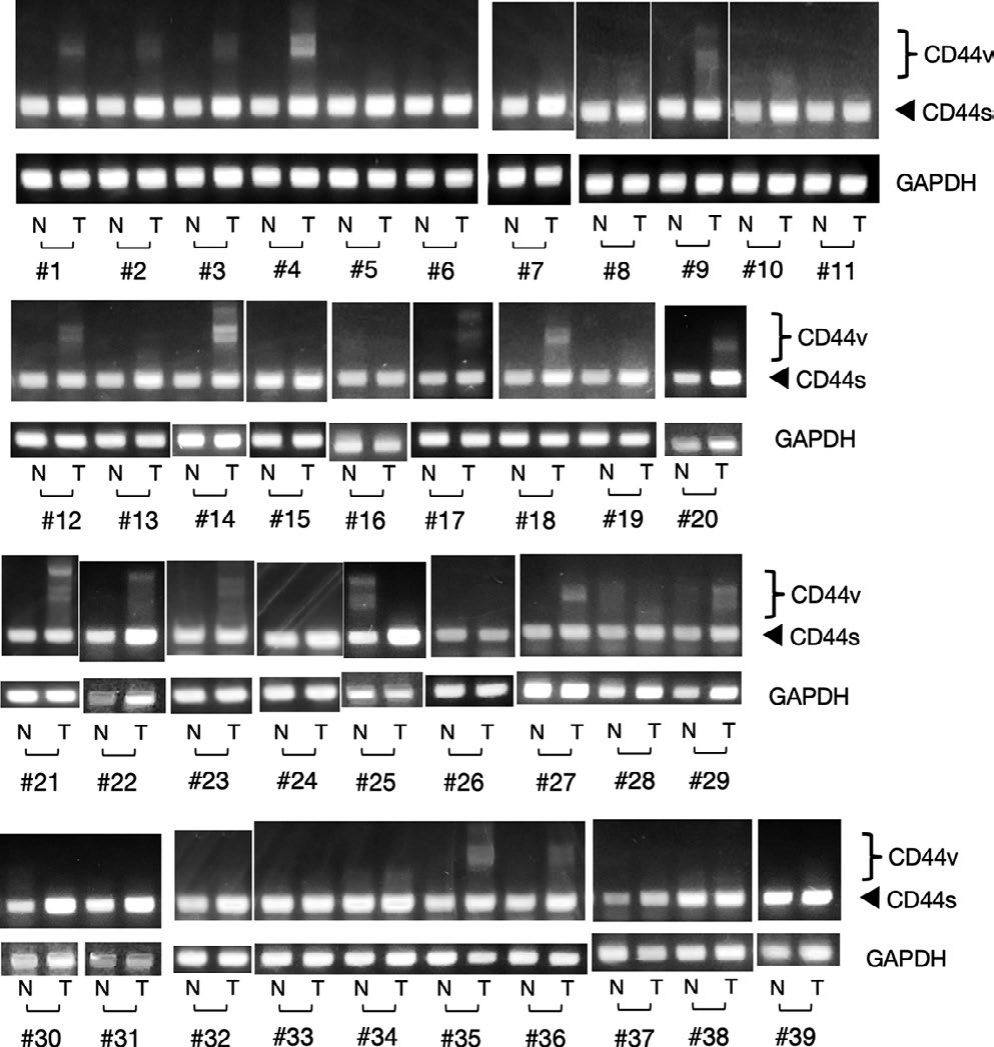
Semi‐quantitative RT‐PCR analysis of*CD44*mRNA in the canine tumours and the normal tissues surrounding the tumours. Total RNA isolated from the canine tumour tissues and the normal tissues surrounding the tumours was subjected to semi‐quantitative RT‐PCR analysis with primers targeting exons 5 and 16 of the canine*CD44*gene. Canine*GAPDH*mRNA was examined as an internal control. The positions of PCR products derived from*CD44v*or*CD44s*(177 bp) are indicated. Samples #1‐3 and 7–11 correspond to breast adenoma, #4‐6 correspond to benign mixed adenoma of the breast, #12 and 14–20 correspond to breast carcinoma, #13 corresponds to malignant mixed tumour of the breast, #21‐22 correspond to oral squamous cell carcinoma, #23‐25 correspond to melanoma, #26‐29 correspond to mastocytoma, #30‐31 correspond to soft tissue sarcoma, #32‐36 correspond to angiosarcoma and #37‐39 correspond to lymphoma. The tumour tissues are indicated by (T), and the normal tissues are indicated by (N)

**FIGURE 2:**
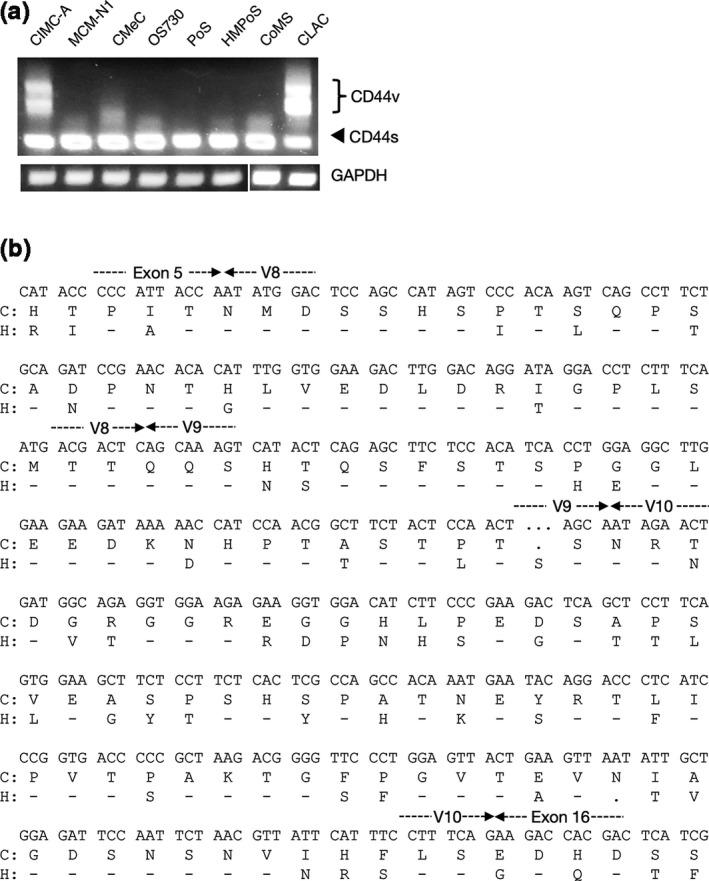
*CD44v8‐10*is expressed in canine tumour cell lines. (a) Total RNA isolated from the canine tumour cell lines was subjected to RT‐PCR analysis with primers targeting exons 5 and 16 of the*CD44*gene as well as the canine*GAPDH*gene. (b) Nucleotide and amino acid sequences of canine CD44v8‐10. The amino acid sequence of human (H) CD44v8‐10 is shown under the canine (c) sequence. Amino acid similarities are indicated by a dash (−), and deletions are indicated by a dot (.). When the human sequence differs from the canine sequence, the amino acid is indicated

We first examined the *CD44* mRNA expression in the canine tumours and the normal tissues surrounding the tumours by semi‐quantitative RT‐PCR. As shown in Figure 1, *CD44s* was expressed in all canine tissues. In contrast, although *CD44v* was expressed in several tumour tissues, it was hardly detected in the normal tissues (Figure 1). The number of cases expressing *CD44* variants was significantly different between the breast tumour tissues and the normal tissues surrounding the tumours (*χ* ^2^ = 10.8, *p* = 0.001; Table 1). Furthermore, we examined the expression of *CD44s* and *CD44v* mRNA in several canine tumour cell lines, including breast tumour (CIMC‐A), melanomas (MCM‐N1 and CMeC), osteosarcomas (OS730, PoS and HMPoS), mastocytoma (CoMS) and lung adenocarcinoma (CLAC) (Figure 2a). The expression of *CD44v* mRNA was observed in CIMC‐A and CLAC cells (Figure 2a). These results indicated that the expression of *CD44* variant isoforms is upregulated in several tumour tissues and cell lines. In addition, we obtained the PCR products from the DNA bands in agarose gels, and examined the nucleotide sequence of the PCR product derived from CIMC‐A by Sanger sequencing. We found that the PCR product derived from CIMC‐A included variant exons 8 to 10, and the nucleotide sequence was consistent with that in the GenBank database (XM_025452466.1) and a previous report on canine *CD44* variant exons (Figure 2b) ( Milde et al., 1994).

**TABLE 1:** The differences of *CD44* variants expression between in the canine tumours and the normal tissues surrounding the tumours

Neoplasm	*CD44v*	Normal	Tumour	*χ* ^2^	*p*
		*n*	*n*		
Benign breast tumour (adenoma + mixed adenoma)	Negative	11	6	6.5	0.042
	Positive	0	5		
Malignant breast tumour (carcinoma + mixed tumour)	Negative	9	4	6.9	0.035
	Positive	0	5		
Total breast tumour (benign + malignant)	Negative	20	10	10.8	0.001
	Positive	0	10		
Oral squamous cell carcinoma	Negative	2	0	0.317	0.167
	Positive	0	2		
Melanoma	Negative	2	2	0.750	0.386
	Positive	1	1		
Mastocytoma	Negative	3	2	0.000	1.000
	Positive	1	2		
Soft tissue sarcoma	Negative	2	2	−	−
	Positive	0	0		
Angiosarcoma	Negative	5	3	0.625	0.429
	Positive	0	2		
Lymphoma	Negative	3	3	−	−
	Positive	0	0		
Total	Negative	37	22	13.6	0.0002
	Positive	2	17		

Note

(−) indicates incalculable.

### 3.2 Overexpression of CD44v8‐10 increases the amount of cellular GSH and prevents canine tumour cell death by H_2_O_2_ or radiation treatment

**FIGURE 3:**
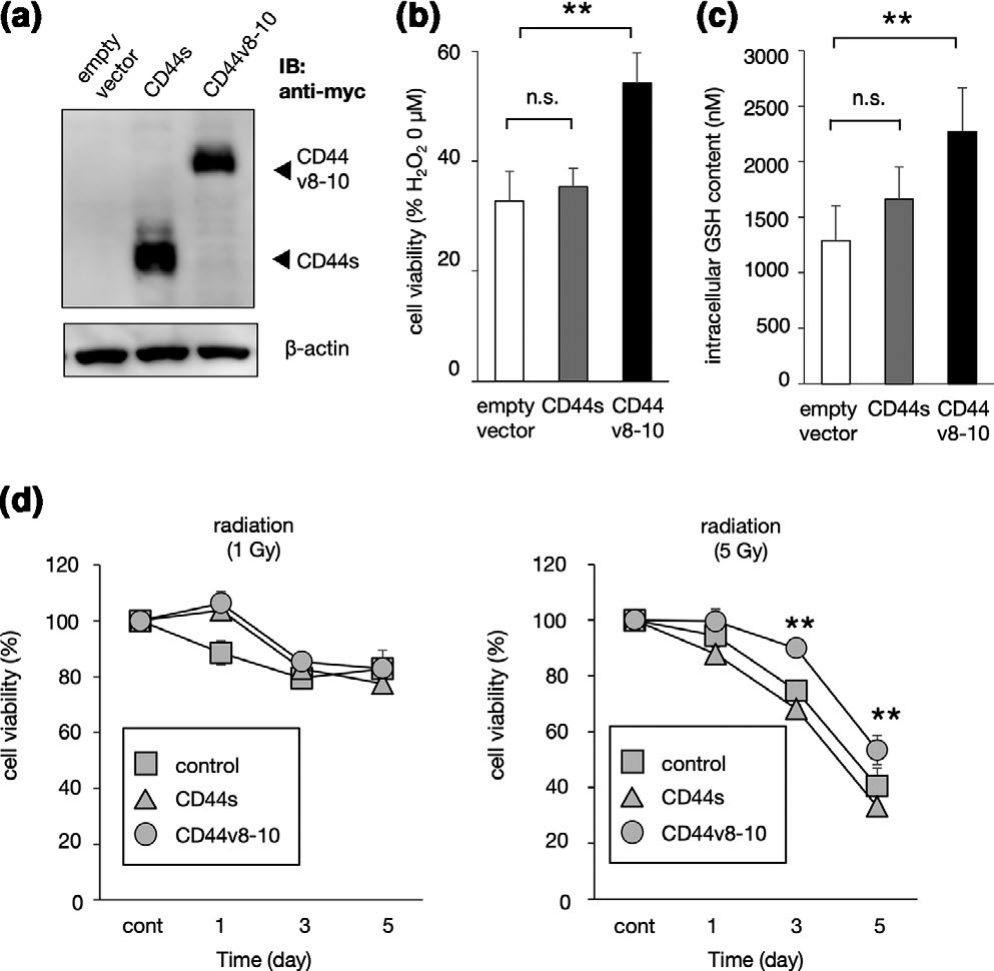
CD44v8‐10 contributes to the resistance of canine breast tumour cells to H_2_O_2_and radiation by promoting the production of GSH. (a) Plasmids expressing canine*CD44s*or*CD44v8‐10*were transfected into the canine breast tumour cell line CIMC‐A. (b) H_2_O_2_‐resistance of CIMC‐A cells transfected with*CD44s*or*CD44v8‐10*. Cell viability values represent the mean ± *SD*relative to the H_2_O_2_‐untreated cells. (c) Cellular GSH content of*CD44*transfectants. (d) Radio‐resistance of*CD44*transfectants. **p < 0.01

To investigate the roles of CD44s and CD44v8‐10 in the ROS‐resistance of canine breast tumour cells, CIMC‐A cells were transfected with *CD44s* or *CD44v8‐10* isoforms, and the resistance to H_2_O_2_ treatment was examined (Figure 3a and b). As shown in Figure 3b, a significant increase in cell viability was observed in the *CD44v8‐10* transfectants, but not in the *CD44s* transfectants. In addition, the intracellular GSH content was increased in the *CD44v8‐10* transfectants when compared with the control and the *CD44s* transfectants (Figure 3c). To further investigate the role of CD44v8‐10 in canine breast tumours, we examined the resistance of these transfectants to radiation treatment. After 3 and 5 days of radiation treatment, the cell viability was significantly higher in the *CD44v8‐10* transfectants than in the control and the *CD44s* transfectants (Figure 3d).

### 3.3 Quantitative RT‐PCR analysis of *xCT* mRNA in canine tumours and the effect of SSZ on H_2_O_2_‐resistance in canine breast tumour cells

**FIGURE 4:**
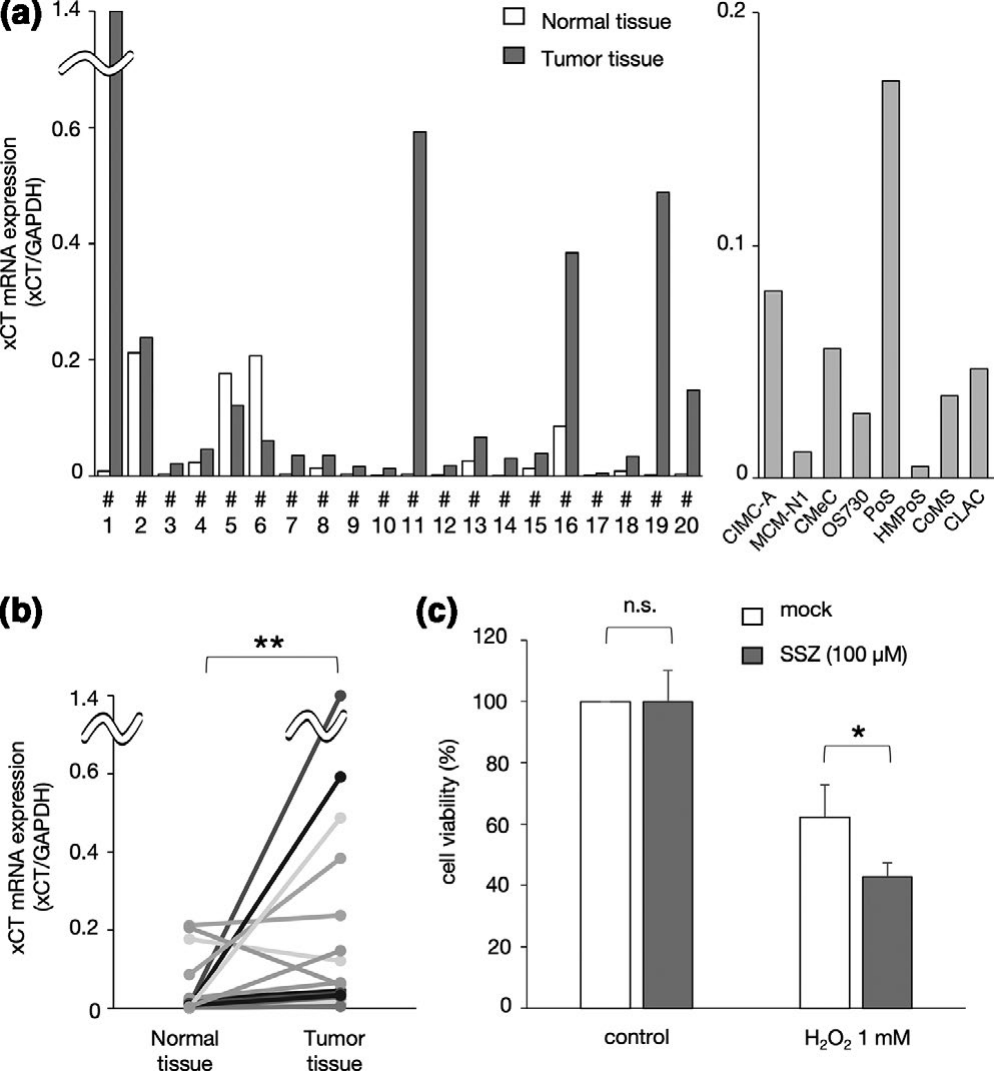
The*xCT*mRNA expression in the canine breast tumour and normal tissues, and effect of xCT inhibition on H_2_O_2_‐resistance of CIMC‐A cells. (a) Quantitative real‐time PCR analysis of*xCT*mRNA in the canine tumours and the normal tissues surrounding the tumours. The sample numbers (#1‐20) are the same as those for the semi‐quantitative RT‐PCR in Figure 1. (b) Comparison of the*xCT*mRNA expression between the canine breast tumour tissues and the normal tissues surrounding the tumours. (c) H_2_O_2_‐resistance of CIMC‐A cells treated with SSZ. Cell viability values represent the mean ± *SD*relative to the H_2_O_2_‐untreated cells. *p < 0.05, **p < 0.01

We next investigated the mRNA expression of *xCT* in the canine breast tumours, the normal tissues surrounding the tumours and the canine tumour cell lines by quantitative real‐time PCR. As shown in Figure 4a and b, the mRNA expression of *xCT* was significantly upregulated in the canine breast tumour tissues as compared to the normal tissues. To investigate whether the inhibition of xCT enhances the cytotoxic effect of H_2_O_2_, we applied SSZ, which is an xCT inhibitor, to CIMC‐A cells. As shown in Figure 4c, the H_2_O_2_ sensitivity of CIMC‐A cells was increased by SSZ treatment (Figure 4c).

## 4 DISCUSSION

Semi‐quantitative RT‐PCR was used to demonstrate that *CD44s* mRNA expression is observed in both normal and tumour canine tissues. CD44s is generally present on the membranes of most hematopoietic and normal epithelial cells, and it binds extracellular matrix ligands, such as hyaluronic acid and fibronectin (Goodison et al., 1999; Ponta et al., 2003; Zöller, 2011). Several studies have reported that CD44s modulates cellular signalling by interacting with various receptor tyrosine kinases, and it induces epithelial‐to‐mesenchymal transition and stem‐like properties in tumour cells (Brown et al., 2011; Mima et al., 2012; Ponta et al., 2003; Zöller, 2011). In contrast, *CD44v* mRNA was expressed only in a subset of canine tumours. Alternative mRNA splicing of *CD44* is mediated by epithelial splicing regulatory protein 1 (ESRP1), (Brown et al., 2011; Jeong et al., 2017; Preca et al., 2015; Yae et al., 2012) and several reports have suggested that the upregulation of ESRP1 is associated with poor prognosis of tumours (Jeong et al., 2017; Yae et al., 2012). A variety of CD44v isoforms generated by ESRP1, such as CD44v6 and CD44v8‐10, have been shown to be involved in multiple cellular functions, including proliferation, adhesion and metastasis (Hirata et al., 2013; Horibe et al., 2018; Ishimoto et al., 2011; Li et al., 2014; Nagano et al., 2013; Ogihara et al., 2019; Patel et al., 2018; Wada et al., 2018; Wu et al., 2015; Yoshikawa et al., 2013). In canine, it has been reported that *CD44v3, v6* and *v7* mRNAs are expressed in B‐cell lymphoma, and the expression of these mRNAs is associated with chemo‐resistance and a poor prognosis (Motegi et al., 2018). However, the relative importance of CD44v isoforms in comparison to the CD44s isoform as a factor for tumour progression remains controversial. Although there have been conflicting reports about the role of CD44 isoforms on tumour phenotypes, we demonstrated that CD44v8‐10, but not CD44s, facilitated the resistance of canine breast tumour cells to H_2_O_2_ and radiation in this study. Namely, CD44v8‐10 was shown to be associated with the xCT transporter, and it contributes to the production of GSH, a major cellular antioxidant (Huang et al., 2005; Lo et al., 2008). Tumour cells are continuously exposed to ROS stress derived from intracellular or extracellular sources; thus, a robust antioxidant system is required for survival and efficient growth (Nagano et al., 2013; Vučetić et al., 2017). Furthermore, upregulation of CD44v has been observed in chemotherapy‐resistant and recurrent tumours, (Hirata et al., 2013; Horibe et al., 2018; Wu et al., 2015; Yoshikawa et al., 2013) suggesting that CD44v plays important roles in tumour progression via the regulation of the cellular antioxidant system.

Moreover, we found that *xCT* mRNA expression was significantly upregulated in the canine breast tumour tissues as compared to the normal mammary gland tissues surrounding the tumour. The upregulation of *xCT* was observed regardless of *CD44v* expression, suggesting that the transcriptional machinery for *xCT* could be activated independently of *CD44v* in canine breast tumours. In human, several studies have reported that the transcription of the *xCT* gene is regulated by transcription factors Nrf2 and ATF4 (Lewerenz & Maher, 2009; Lewerenz et al., 2012; Sasaki et al., 2002). Translation of the ATF4 protein is enhanced by multiple types of stresses, including oxidative stress; thus, ATF4 is thought to be involved in the resistance of tumours to chemotherapy via the regulation of *xCT* expression (Lewerenz & Maher, 2009; Lewerenz et al., 2012). To investigate the function of xCT in the resistance of tumours to chemotherapy, several studies have employed SSZ as an inhibitor of xCT (Gout et al., 2001; Ishimoto et al., 2011; Ma et al., 2015; Ogihara et al., 2019; Wada et al., 2018; Yoshikawa et al., 2013). Indeed, previous studies showed that SSZ treatment enhanced the effect of several chemotherapies (Gout et al., 2001; Ishimoto et al., 2011; Ogihara et al., 2019; Wada et al., 2018; Yoshikawa et al., 2013). Consistent with the previous reports, we showed that SSZ treatment enhanced the cytotoxic effect of H_2_O_2_ in canine breast tumour cells, suggesting that xCT plays an important role in the therapy resistance of canine tumours as well as human tumours.

In conclusion, our present findings suggested that CD44v and xCT are expressed in several canine tumours, and they contribute to the resistance of tumour cells to oxidative stress and radiation. CD44v8‐10‐overexpressing canine breast tumour cells were shown to produce GSH at a higher level than CD44s‐overexpressing cells. Thus, tumour‐specific antioxidant systems may be a potential target for the chemotherapy and radiotherapy of canine tumours.

Authorship: A.T. has contributed to the analysis of data and drafting the manuscript. K.K., H.T and M.T. has contributed to the acquisition of data. H.S. has contributed to the conception and design and drafting of the manuscript.

## CONFLICT OF INTEREST

The authors declare no conflicts of interest for this article.

## AUTHOR CONTRIBUTION

**Atsushi Tanabe:** Investigation; Writing‐original draft. **Kento Kimura:** Investigation. **Hana Tazawa:** Investigation. **Takuya Maruo:** Formal analysis; Investigation. **Masayuki Taguchi:** Investigation; Resources. **Hiroeki Sahara:** Conceptualization; Funding acquisition; Project administration; Writing‐review & editing.

### PEER REVIEW

The peer review history for this article is available at https://publons.com/publon/10.1002/vms3.397.

## Data Availability

The data that support the findings of this study are available from the corresponding author upon reasonable request.
